# Lumen-apposing metal stent fixation using a defect-closure system for the management of a case of afferent limb syndrome

**DOI:** 10.1055/a-2764-4656

**Published:** 2026-01-20

**Authors:** Marco Spadaccini, Valeria Poletti, Ludovico Alfarone, Matteo Colombo, Giacomo Marcozzi, Alessandro Fugazza, Alessandro Repici

**Affiliations:** 1 9268 Department of Gastroenterology, IRCCS Humanitas Research Hospital, Rozzano, Milan, Italy; 2 437807 Department of Biomedical Sciences, Humanitas University, Pieve Emanuele, Milan, Italy

Endoscopic ultrasound (EUS)-guided drainage is considered the first-line approach for afferent limb syndrome 1 . We report the case of a 76-year-old woman with pancreatic adenocarcinoma who underwent pancreaticoduodenectomy in 2022 and later developed abdominal disease progression ( Video 1 ).

A patient with afferent limb syndrome after pancreaticoduodenectomy was treated with EUS-GE and anchored LAMS placement. Subsequently, EDGI with a second LAMS across the proximal stricture achieved complete clinical and laboratory resolution. EDGI, EUS-directed transgastric intervention; EUS-GE, endoscopic ultrasound-guided gastroenterostomy; LAMS, lumen-apposing metal stent.Video 1

The patient presented with two malignant strictures of the afferent loop, which was markedly dilated, resulting in biliary tract dilation, jaundice, and early cholangitis. An initial endoscopic ultrasound-guided gastroenterostomy (EUS-GE) was performed with deployment of a 15 mm × 10 mm lumen-apposing metal stent (LAMS; Hot Axios, Boston Scientific) between the stomach and the afferent loop at the segment best visualized endosonographically, located between the two strictures. As the loop remained dilated down to the proximal stricture, its clinical significance was initially uncertain; therefore, drainage was directed to the only accessible tract.

**Fig. 1:**
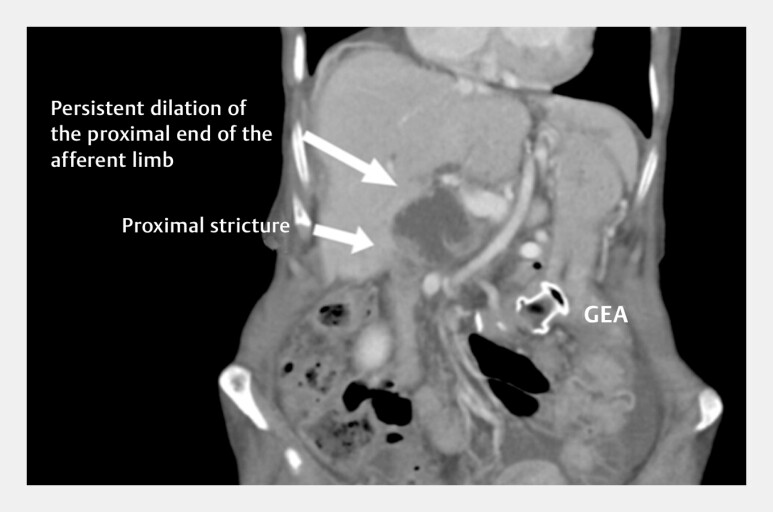
Persistent dilation of the biliary tree and the proximal end of the afferent limb, despite the technically successful placement of EUS-GE. EUS-GE, endoscopic ultrasound-guided gastroenterostomy.

Although technically successful, the patient’s condition worsened 3 days later with the rapid onset of cholangitis. Computed tomography confirmed the relevance of the proximal stricture, requiring additional intervention ( Fig. 1 ). To prevent LAMS migration, the stent was anchored to the gastric wall using an X-Tack Endoscopic HeliX Tacking System (Boston Scientific). A 10.8 mm endoscope was then advanced through the LAMS into the afferent loop, reaching the proximal stricture near the biliary tract. Through an EUS-directed transgastric intervention (EDGI), a second 15 mm × 15 mm LAMS was deployed across the stricture, achieving the complete resolution of symptoms and normalization of laboratory values.

This case demonstrates the successful stepwise endoscopic management of afferent limb syndrome in complex post-surgical anatomy 2 . While direct EUS-guided drainage was not feasible, combining EUS-GE with EDGI offered an effective solution. Fixation of the initial LAMS using a defect-closure system minimized the dislodgement risk 3 and allowed safe EDGI within days of the first procedure, a strategy potentially amenable to single-step use.

Endoscopy_UCTN_Code_TTT_1AS_2AH
